# Variability in the pediatric intensivists' threshold for withdrawal/limitation of life support as perceived by bedside nurses: a multicenter survey study

**DOI:** 10.1186/2110-5820-1-31

**Published:** 2011-08-08

**Authors:** Colleen S Gresiuk, Ari R Joffe

**Affiliations:** 1 University of Alberta, Stollery Children's Hospital, 8440 112 Street, Edmonton, Alberta, T6G 2B7, Canada; 2 The John Dossetor Health Ethics Center, 8440 112 Street; Edmonton, Alberta, T6G 2B7, Canada

## Abstract

**Background:**

We hypothesized that bedside nurses perceive significant variability in the pediatric intensivist thresholds for approaching a family about withdrawal/limitation of life-sustaining therapy.

**Methods:**

All nurses working in four university-affiliated medical-surgical pediatric intensive care units staffed by 11, 7, 6, and 5 intensivists with 36, 18, 10, and 8 beds were sent three mailings of a survey asking questions about intensivist decisions for withdrawal/limitation of life-sustaining therapy. Responses were tabulated; chi-square compared results among centers; a *p *< 0.05 after Bonferroni correction was significant.

**Results:**

The response rate was 205 of 415 (49%); 152 of 205 (74%) disagreed with the statement that each of the intensivists had the same threshold for approaching a family to suggest withdrawal/limitation of life-sustaining therapy, with no significant difference between centers. Also, 110 of 205 (54%) and 119 of 205 (58%) disagreed with the statement that each intensivist has the same threshold of the patient's chance for survival or projected quality of life when making a decision to withdraw/limit life-sustaining therapy with no significant difference between centers. The threshold to suggest withdraw/limit life-sustaining therapy based on chance of survival or projected quality of life differs between intensivists by at least 10% according to 113 of 184 (61%) and 121 of 184 (66%) nurses; the two larger centers had significantly higher difference among intensivists for projected quality of life. Fifty-five of 200 (27%) disagreed with the statement that they would have equal confidence in each intensivist accepting a recommendation for withdrawal/limitation of life-sustaining therapy for their own child, with no difference between centers.

**Conclusions:**

Bedside pediatric intensive care unit nurses in this multicenter Canadian study perceive wide variability in intensivist thresholds for approaching a family to suggest withdrawal/limitation of life-sustaining therapy.

## Introduction

Most deaths in Pediatric Intensive Care Units (PICU) follow withdrawal or limitation of life-sustaining treatments (W/L) [1], and the number has increased in recent years [2-5]. This is considered ethically permissible in the context of legally incompetent minors when the proportionately greatest benefits of treatment are outweighed by the harms of treatment [6]. The principle of patient autonomy allows a competent patient or their surrogate to refuse or stop any lifesaving treatment when it is what the patient would wish for their care [6,7]. In contrast, for children, the decision is usually made in discussion with the parents to arrive at a treatment plan that is in the best interests of the child.

There is limited research to determine how these decisions are made. When presented with hypothetical patient scenarios, the thresholds for W/L vary significantly between intensivists in both adult [8] and pediatric [9] intensive care units (ICU). One study showed that medical residents perceive a difference in thresholds between ICU attending physicians when making these decisions [10]. The bedside nurses' perception is another and, arguably, potentially a more realistic independent observer-based reflection of these decisions than asking intensivists to respond to hypothetical scenarios. To our knowledge, there is no multicenter study that evaluates the threshold of pediatric intensivists in decisions to W/L in everyday practice.

We previously reported a single-center survey of PICU nurses where respondents perceived significant variability in intensivist thresholds for approaching a family to suggest W/L [11]. Our objective was to determine whether this is a reproducible finding in Canadian PICUs. Wide decisional variability is not desirable; it raises significant concern considering the implication of W/L decisions for patient mortality. We find that bedside PICU nurses in this multicenter Canadian study perceive wide variability in intensivist thresholds for approaching a family to suggest W/L.

## Methods

### Questionnaire administration

This study was a survey of PICU nurses' opinions regarding their experience with W/L and do not resuscitate (DNR) orders. Each staff nurse in four university-affiliated, tertiary, medical-surgical Canadian PICUs was delivered the survey in 2009. The four PICUs were in Alberta and Ontario, each staffed by 11, 7, 6, and 5 full-time intensivists with 36, 18, 10, and 8 beds. A cover letter was included asking the nurse to fill in the survey and return it in the self-addressed envelope. A second and third delivery of the survey was done at 4- to 8-week intervals to nonresponders, and all responses were considered received by the end of 2009.

The cover letter stated:

"As you know, unfortunately, children may die from critical illness in the PICU. Often the decision is made to limit therapy (including a DNR or "do not resuscitate" order), or withdraw therapy to allow a patient to die when the harms of treatment outweigh any potential benefits. This is a value-laden decision, based on an assessment of prognosis and quality of life (QOL), and heavily influenced by religious and personal beliefs. You are one of the 'front line' workers in the PICU who sees these decisions made and the effect of them on staff, patient, and families. We have designed this survey as an attempt to determine your perspective on this process of 'ethical decision making in the PICU' as a bedside nurse.... Your responses are voluntary and confidential...return of the survey implies consent to participate."

The study was approved by our university health research ethics board.

### Questionnaire development

The development of the survey has been described previously [11]. We searched MEDLINE from 1966 to 2004 for articles on W/L from children in a PICU, using search terms, including "withholding treatment," "resuscitation orders," "pediatrics," and "child," and found no reports of a similarly designed study. We wanted the three-page survey (Additional File 1) to be simple and focused. Therefore, to generate the items for inclusion in the questionnaire, we focused the questions specifically on any perceived differences in the threshold to suggest W/L and DNR by each pediatric intensivist. There are no written guidelines for end-of-life decision-making in the PICUs apart from those published by the American Academy of Pediatrics and the Canadian Pediatric Society, which intensivists in Canada are expected to follow [6,12]. In general, these decisions are made jointly by the parents and attending intensivist at the time, with meetings that include the bedside nurse, and commonly pastoral care and social work.

Questions utilized a five-point Likert scale: strongly agree (SA), agree (A), neutral, disagree (D), or strongly disagree (SD). Two questions did not use this Likert scale; these questions stated that "the threshold to suggest W/L based on chance of survival (or based on projected QOL) differs among intensivists by: < 1%, 5%, 10%, 15%, or ≥ 20%.

To ensure clarity, perceived reality of the situations presented, validity, and ease of completion of the questionnaire, initial pilot testing was done by having the survey completed by six PICU nurses, followed by a semistructured interview. All found the survey to be understandable, easy to follow, not difficult or confusing, and were confident that their responses reflected their intended answers. The survey asked nurses to make a subjective judgment about intensivist thresholds to suggest W/L. We chose this subjective measure to describe nursing perception of practice variations. There is no standard threshold defined; indeed, a set standard is not desirable, because it could not be expected to take into account all of the myriad of considerations of each individual case, and whose (patient, parent, physician, nurse, ethicist, etc.) values would define it is problematic. The pilot testing indicated that the respondents understood this subjective concept of "threshold."

### Statistics

Anonymous data were entered into a computer database (Microsoft^® ^Excel; Microsoft Corp, Redmond, WA). The proportion of respondents with different answers was tabulated. We compared responses between the four PICUs by grouping responses into SA/A, neutral, and D/SD. In addition, two subgroups in the pooled data were identified before survey distribution to reflect the level of PICU experience: those working in PICU < 5 years vs. ≥ 5 years, and those who had attended < 5 vs. ≥ 5 family meetings. The responses in the PICUs and in these subgroups were compared using the Chi-square statistic, with *p *< 0.05 accepted as significant after Bonferroni adjustment for multiple comparisons.

## Results

### Survey respondents

Of 415 surveys delivered, 205 (49%) were returned. The respondents' demographics are shown in Table 1.

**Table 1:** Demographics of the survey respondents

Question	A (n = 37)	B (n = 35)	C (n = 49)	D (n = 84)	Total (n = 205)
**Response rate**					*p *< 0.001*
Distributed	46	50	70	249	415
Returned	37 (80%)	35 (70%)	49 (70%)	84 (34%)	205 (49%)
Leave of absence	0 (0%)	5 (14%)	0 (0%)	0 (0%)	5 (2%)
**Sex**					*p *= 0.11
Male	3 (8%)	0 (0%)	0 (0%)	5 (6%)	8 (4%)
Female	34 (92%)	35 (100%)	49 (100%)	79 (94%)	197 (96%)
**Age (yr)**					*p *= 0.001
20-30	6 (16%)	14 (40%)	32 (65%)	29 (35%)	81 (40%)
30-40	11 (30%)	12 (34%)	12 (24%)	24 (29%)	59 (29%)
40-50	12 (32%)	7 (20%)	4 (8%)	23 (27%)	46 (22%)
50-60	7 (19%)	2 (6%)	1 (2%)	7 (8%)	17 (8%)
60+	0 (0%)	0 (0%)	0 (0%)	0 (0%)	0 (0%)
**Years in practice**					*p *< 0.001
< 5	5 (14%)	10 (29%)	35 (71%)	25 (30%)	75 (37%)
5-10	9 (24%)	11 (31%)	6 (12%)	15 (18%)	41 (20%)
11-15	2 (5%)	5 (14%)	2 (4%)	16 (19%)	25 (12%)
16-20	5 (14%)	5 (14%)	3 (6%)	8 (10%)	21 (10%)
> 20	16 (43%)	4 (11%)	3 (6%)	20 (24%)	43 (21%)
**Years in PICU**					*p *< 0.001
< 5	11 (30%)	20 (57%)	41 (84%)	34 (40%)	106 (52%)
5-10	7 (19%)	10 (29%)	3 (6%)	23 (27%)	43 (21%)
11-15	3 (8%)	3 (9%)	2 (4%)	6 (7%)	14 (7%)
16-20	7 (19%)	1 (3%)	2 (4%)	8 (10%)	18 (9%)
> 20	9 (24%)	1 (3%)	1 (2%)	13 (15%)	24 (12%)
**No. of family meetings attended**					*p *< 0.001
< 5	13 (35%)	12 (34%)	38 (78%)	31 (37%)	94 (46%)
5-10	6 (16%)	8 (23%)	4 (8%)	16 (19%)	34 (17%)
11-15	5 (14%)	6 (17%)	1 (2%)	8 (10%)	20 (10%)
> 15	13 (35%)	9 (26%)	6 (12%)	29 (35%)	57 (28%)

*p values refer to the difference in the demographic factor between the four institutions.

### The intensivist's role in decisions

The responses to questions about the threshold to approach a family, the family contribution to decisions, and unilateral decisions are shown in Table 2. Most respondents (152/205, 74%) did not believe that each of the intensivists have the same threshold for approaching a family to suggest a W/L or DNR order, and many (68/205; 33%) did not believe that each intensivist allows the same amount of family contribution to these decisions.

**Table 2:** Responses to the questions about the intensivist's role in making withdrawal/limitation of therapy decisions

Survey statement	Strongly agree/agree	Neutral	Disagree/strongly disagree
Each of the PICU intensivists has the same threshold for approaching a family to suggest a W/L or DNR (n = 205).	21 (10%)	32 (16%)	119 (74%)
The threshold is too high with some intensivists (i.e., the discussion occurs too late) (n = 205).	149 (73%)	29 (14%)	26 (13%)
The threshold is too low with some intensivists (i.e., the discussion occurs too early) (n = 204).	33 (16%)	40 (20%)	131 (64%)
Each intensivist allows the same amount of family contribution to the decision regarding W/L or DNR (n = 203).	79 (39%)	56 (28%)	68 (33%)
Too much family influence is allowed with some intensivists (n = 204).	98 (48%)	42 (21%)	64 (31%)
Too little family influence is allowed with some intensivists (n = 204).	34 (17%)	61 (30%)	109 (53%)
A PICU intensivist has W/L without having a discussion with the family (n = 202).	10 (5%)	18 (9%)	174 (86%)
This occurs often (n = 205).	10 (5%)	29 (14%)	166 (81%)
Each intensivist has the same threshold for W/L without having a discussion with the family (n = 201).	22 (11%)	47 (23%)	132 (66%)

W/L = withdrawal or limitation of life support treatment; DNR = do not resuscitation order; PICU = pediatric intensive care unit

**Figure 1:**
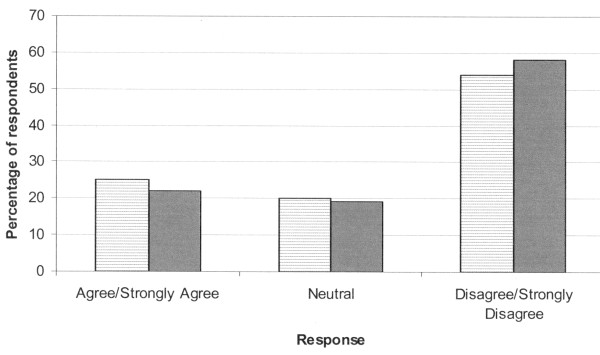
**Response to the statement**: "Each intensivist has the same threshold of the patient's chance for survival [or, projected quality of life] when making a decision to limit/withdraw therapy." Chance for survival: hatched bars; quality of life: solid bars.

In two questions, it was stated that "each intensivist has the same threshold of the patient's chance for survival [or, projected QOL] when making a decision to W/L." Of respondents, the majority disagreed with these statements (Figure 1). The next two questions stated: "The threshold to suggest to W/L based on chance of survival [or, projected QOL] differs between intensivists by: < 1%, 5%, 10%, 15%, or ≥ 20%." For the question based on chance of survival, respondents answered < 1% in 20 (10%), 5% in 52 (25%), 10% in 61 (30%), 15% in 28 (14%), and ≥ 20% in 24 (12%). For the question based on projected QOL, respondents answered < 1% in 18 (9%), 5% in 45 (22%), 10% in 56 (27%), 15% in 32 (16%), and ≥ 20% in 33 (16%). The threshold to suggest to W/L based on chance of survival differed between intensivists by at least 10% for 113 of 185 (61%) and by at least 15% for 52 of 185 (28%) of respondents. The threshold to suggest to W/L based on QOL differed between intensivists by at least 10% for 121 of 184 (66%) and by at least 15% for 65 of 184 (35%) respondents. Although subjective, we believe that these may be meaningful differences in outcome for some patients.

Few statistically significant differences between PICUs were found in the responses to questions (Table 3). In each case, the two smaller PICUs suggested more consistency in not allowing too much family contribution, not often having unilateral decisions, and the threshold of projected QOL in making decisions.

**Table 3:** Questions where there were statistically significant differences in responses between the four pediatric intensive care units

Question	A (n = 37)	B (n = 35)	C (n = 49)	D (n = 84)	Total (n = 205)	*p *value
Each intensivist allows the same amount of family contribution to the decision regarding W/L or a DNR. Too much family influence is allowed with some intensivists (n = 204).						< 0.001
SA/A	11 (30%)	11 (31%)	34 (69%)	42 (50%)	98 (48%)	
N	9 (24%)	5 (14%)	10 (20%)	18 (21%)	42 (21%)	
D/SD	17 (46%)	19 (54%)	5 (10%)	23 (27%)	64 (31%)	
A PICU intensivist has W/L without having a discussion with the family. This occurs often (n = 205).						< 0.001
SA/A	1 (3%)	0 (0%)	0 (0%)	17 (20%)	10 (5%)	
N	0 (0%)	0 (0%)	7 (14%)	22 (26%)	29 (14%)	
D/SD	36 (97%)	35 (100%)	42 (86%)	53 (63%)	166 (81%)	
The threshold to suggest W/L based on projected QOL differs among intensivists by:						0.003
< 1%	6 (16%)	7 (20%)	0 (0%)	5 (6%)	18 (9%)	
5%	10 (27%)	10 (29%)	9 (18%)	16 (19%)	45 (22%)	
10%	13 (35%)	9 (26%)	12 (24%)	22 (26%)	56 (27%)	
15%	4 (11%)	0 (0%)	13 (27%)	15 (18%)	32 (16%)	
> 20%	3 (8%)	5 (14%)	12 (24%)	13 (15%)	33 (16%)	
Blank	1 (3%)	4 (11%)	3 (6%)	13 (15%)	21 (10%)	

All comparisons by Chi-square test

W/L = withdrawal or limitation of life support treatment; DNR = do not resuscitate order; QOL = quality of life; SA/A = strongly agree or agree; N = neutral; D/SD = disagree or strongly disagree; N/A = not answered

### The hypothetical "nurse's own child" scenario

**Figure 2:**
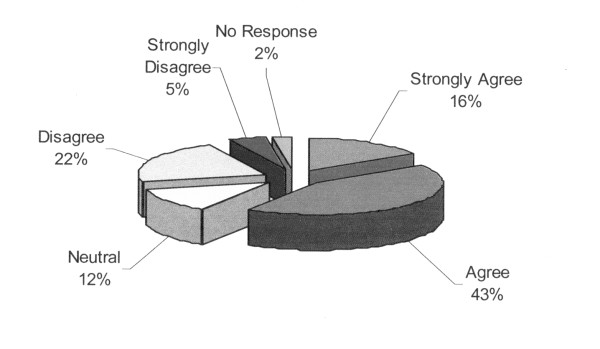
**Response to the question**: "Assume your child was in the pediatric intensive care unit and the intensivist on service approached you to recommend a limiting/withdrawing life support or 'do not resuscitate' order. You would have equal confidence accepting this recommendation from each intensivist."

A significant minority of respondents (55/205, 27%) disagreed with the statement that, for their own child in the PICU, they would have equal confidence in accepting a recommendation for W/L or DNR from each intensivist (Figure 2). Similarly, 93 of 205 (45%) responded that they would have confidence in the intensivist's opinion to W/L only in certain situations (Additional File 2). For these questions, there were no statistically significant differences between centers.

With this last question, the survey asked to "please explain." Comments were written by only 77 of 205 (38%) respondents. On review of the written comments, we determined that all could be classified into two themes: the confidence level in the intensivists' recommendations for W/L (n = 57), and general comments on how these decisions should be made (n = 35). Of the 57 comments regarding confidence level in the intensivist recommendations, comments suggested total confidence in 24 (42%), confidence in certain situations only in 23 (40%) [some wanted to review all the tests done for themselves (n = 5, 22%), and some suggested that the intensivist and the nurse's view of QOL may differ (n = 10, 43%)], and lack of confidence in 10 (17%) [some suggested that they would have confidence in an individual intensivist only (n = 6, 60%), and some claimed that they have seen disagreement amongst intensivists leading them to doubt individual opinions (n = 3, 30%)]. Of the 33 general comments, the comments included a need for multidisciplinary team and consultant involvement in these decisions in 9 (27%), the decision to suggest W/L is delayed too long in 16 (48%), and other comments in 9 (27%). Examples of written comments are shown in Additional File 3.

### Differences between respondent subgroups

Only one question had statistically significant differences in responses in the two subgroups: more experienced nurses--with ≥ 5 years of PICU experience or ≥ 5 family meetings--were more likely to respond D/SD that intensivists have W/L without discussion with the family (Table 4). There were remarkably similar results between the subgroups in the questions asking for the threshold for W/L based on chance of survival or QOL (all *p *> 0.9).

**Table 4:** Response to the survey questions in the prespecified subgroups of nurses

Question	Subgroup	SA/A	N	D/SD	Blank	*p *value
A PICU intensivist has W/L without having a discussion with the family.						

	< 5 yr PICU (n = 106)	4 (4%)	17 (16%)	83 (78%)	2 (2%)	0.001
	> 5 yr PICU (n = 99)	6 (6%)	1 (1%)	91 (92%)	1 (1%)	
	< 5 meetings (n = 94)	2 (2%)	17 (18%)	73 (78%)	2 (2%)	< 0.001
	> 5 meetings (n = 111)	8 (7%)	1 (1%)	101 (91%)	1 (1%)	

All questions not included in the table demonstrated no significant difference in responses between subgroups. All comparisons are by Chi-square test

W/L = withdrawal or limitation of life support treatment; DNR = do not resuscitate order; SA/A = strongly agree or agree; N = neutral; D/SD = disagree or strongly disagree; N/A = not answered

## Discussion

To our knowledge, this is the first published multicenter report to examine bedside PICU nurses' perception of variability in the pediatric intensivists' thresholds for approaching a family about W/L or DNR decisions. We found that these nurses do perceive significant variability among the intensivists. Of respondents, only 21 (10%) agreed with the statement that each of the intensivists had the same threshold for approaching a family to suggest W/L. Only 52 (25%) and 46 (22%) respondents agreed with the statement that each intensivist has the same threshold of the patient's chance for survival or projected QOL when making a decision to W/L. Most nurses perceived that the difference between intensivists in the threshold to suggest W/L based on chance of survival (n = 113, 61%) or projected QOL (n = 121, 66%) differs by at least 10%. This perception may explain the response to the question of the confidence that the nurse would have in the intensivist's opinion if it concerned the nurse's own child: only 121 (59%) agreed with the statement "you would have equal confidence accepting this recommendation [for W/L] from each intensivist." The nurses' written comments about confidence level (n = 57) support this conclusion: 42% suggested total confidence in the recommendation of each intensivist, 40% suggested confidence in only certain situations, and 17% suggested a lack of confidence. These results confirm and generalize our previous single-center study using the same survey instrument [11].

Our results are compatible with other studies that used different methodologies. The thresholds for W/L vary significantly between intensivists in both adult and pediatric studies where hypothetical patient scenarios are presented [8,9]. Medical residents perceive a difference in thresholds among their attending physicians in making W/L decisions [10]. Hospital characteristics are associated with the use of DNR orders, even after accounting for differences in patient characteristics; indeed, a tenfold difference in standardized rates of DNR across counties in California may reflect different institutional cultures [13]. In Europe, the frequency of DNR and W/L decisions varies markedly between and within countries [14,15].

The nurse-perceived variability in physician thresholds to suggest W/L in our study is of significant concern considering the implication of W/L for patient mortality. A multicenter Canadian study found that of the 341 adult patients who were assessed by a physician on at least one occasion to have a probability of ICU survival of < 10%, 99 (29%) survived the ICU [16]. Even for those where this prediction was made on at least three occasions, the actual survival was 27 of 120 (22.5%). For patients with clinician predicted survival of 10-40%, the actual survival was 79.3%. When the physician predicted a survival of < 10%, patients were more likely to have withdrawal of life support (including ventilation, inotropes, and dialysis), and this prediction more powerfully predicted ICU mortality than illness severity, evolving or resolving organ dysfunction, and use of inotropes or vasopressors, and predicted mortality more strongly for patients who had no stated preferences regarding W/L and who had less severe organ dysfunction [16]. The withdrawal of mechanical ventilation was predicted by the physician's prediction of the likelihood of patient survival in ICU of < 10%, and not by patient age, prior functional status, severity of illness, or severity of organ dysfunction [17]. Other studies have found that there is large variability in the accuracy of prognostication by intensivists [18]. This can and does lead to self-fulfilling prophesies in predicting outcomes [19]. In the Canadian multicenter study, 3.6% of patients who had withdrawal of mechanical ventilation in anticipation of death were discharged home [17], and in an international ICU adult study the proportion of hospital survivors who had W/L decisions ranged from 2.4-30.3% [20].

The Task Force on Values, Ethics, and Rationing in Critical Care (VERICC) suggests that rationing decisions based on clinician judgment are "particularly susceptible to unethical subjectivity and bias" [21]. Examples cited include rationing decisions made based on: age, pre-illness employment status, the political power of the surgical services, race, iatrogenic complications, families who the physician knows or likes particularly well, and families who are more demanding [21]. They suggest that "when clinicians withhold interventions based on their interpretation of the standard of care...it becomes clear that a potentially beneficial intervention is being withheld for reasons other than the best interests of the patient [21]." Although we did not ask what factors the nurses believed influenced the variability among intensivists in our survey, it is likely that some of the conscious or unconscious [22] biases mentioned influence the individual intensivist's judgments about chance of survival and QOL. The nurses' comments suggested that some have confidence in only individual intensivists and some have a different view on QOL than certain intensivists. A "shared decision-making" model has been suggested for end-of-life decisions [23]. In this model, one component is a physician's recommendation. Concerns with this model include that the power differential between physician and patient and physician's personal biases may both unduly influence the decision [23,24]. This study suggests that the concern about personal bias may be very real.

There has been debate about whether physicians can make unilateral decisions about W/L and DNR orders [25-30]. These decisions involve value judgments about the chance of survival and the worth of differences in QOL without any uniform consensus [29-31]. There are very few circumstances where one can invoke the "futility" argument [29-32]. The recently recommended shared decision-making model explicitly suggests that clinicians discuss prognostic uncertainty with family decision makers, and empiric data show that most surrogates desire this be acknowledged [33,34]. There are "no objective incontrovertible metrics" for prognostication and "no clinician is omniscient; no clinician is infallible; and the clinician [should not] prioritize his [or his perception of the patient's] values...." [28]. If there are varying knowledge, biases, values, and recent experience among intensivists, the end-of-life decision making "must not depend on luck of the draw: who is in the emergency department or intensive care unit that night" [28]. Our survey found that many nurses perceive that intensivists have different judgments regarding what chance of survival or QOL is worth pursuing.

Strengths of this study include the multicenter representation, the reasonable response rate (204/415 subjects; 49%), the survey development methods, including the simple focused nature of the questions, and the favorable pilot testing. Most respondents were highly experienced, having been in practice for ≥ 5 years in 63% and working in the PICU for ≥ 5 years in 48%. The similar results compared with studies that used different methodologies and our previous single-center study enhanced the generalizability of the findings.

Limitations of this study include: lack of open-ended questions allowing respondents to expand on their intended answers, and the possible discrepancies between perceived and actual practice of the intensivists. In addition, factors that may explain the heterogeneity of responses were not available. It is unclear whether all of the intensivists differ in their threshold or whether a subgroup of intensivists are perceived to be more homogenous in their threshold. The survey required subjective recalled perceptions for responses, even in questions that attempted to quantify the amount of variability. It is difficult to know exactly what, for example, a 10% difference in QOL means to the respondents. Finally, testing for statistical differences in responses among institutions should be interpreted with caution given our small sample size and inadequate power to rule out differences. However, we believe that these limitations would not affect the main conclusion of this study.

## Conclusions

This multicenter study demonstrates that bedside PICU nurses perceive wide variability in the intensivist's threshold for approaching a family to suggest W/L; this variability includes different thresholds of the chance of survival and projected QOL. This finding has significant implications for how end-of-life decisions, particularly unilateral decisions, are made in a PICU. We suggest that intensivists need to be aware of this nursing perception and to consider seriously its implications for their own decision making. The intensive care that a child is offered may depend to a large degree on the physician in charge, which may affect a patient's mortality and palliative care decisions. Further study is required to determine ways to improve consistency in end-of-life practice.

## Abbreviations

A/SA: agree or strongly agree; D/SD: disagree or strongly disagree; DNR: do not resuscitation; PICU: pediatric intensive care unit; QOL: quality of life; W/L: withdrawal/limitation of life-sustaining therapy.

## Competing interests

The authors declare that they have no competing interests.

## Authors' contributions

AJ drafted the first version of the manuscript. CG and AJ made substantial contributions to conception and design of the study, acquisition of data, and analysis and interpretation of data, have been involved in revising the manuscript critically for important intellectual content, and have given final approval of the version to be published.

## Supplementary Material

Additional file 1 **The survey instrument**. Table Click here for file

Additional file 2 **Response to the question: For your own child, "you would have confidence in the intensivist's opinion to limit/withdraw life support only in certain situations**." Figure Click here for file

Additional file 3 **Representative written comments to the instruction "please explain" after questions about confidence in intensivist decisions for the nurse's hypothetical own child**. Table Click here for file
