# Supplementary material for: Variability in the pediatric intensivists' threshold for withdrawal/limitation of life support as perceived by bedside nurses: a multicenter survey study
Source: Ann Intensive Care. 2011 Aug 8;1:31. doi: 10.1186/2110-5820-1-31 (PMC3224498; doi:10.1186/2110-5820-1-31)
Supplement: Additional file 1 — The survey instrument. Table [file 2110-5820-1-31-S1.DOC]

**Variability in the pediatric intensivists’ threshold for withdrawal/limitation of life support as perceived by bedside nurses: a multicenter survey study**

**Journal Name:** Annals of Intensive Care

**Authors:** Colleen S Gresiuk RN, BScN^1^; and Ari R Joffe MD, FRCPC^1,2^

**Affiliations:** ^1^University of Alberta, ^1^Stollery Children’s Hospital, and ^2^The John Dossetor Health Ethics Center; Edmonton, Alberta, Canada.

**Corresponding author:** Ari R Joffe MD; Email: [ari.joffe@albertahealthservices.ca](mailto:ari.joffe@albertahealthservices.ca)

**Additional File 1: The survey instrument.**

# Page 1: Demographics

# 1. Sex:

# Male

# Female

# 2. Age

# 20-29

# 30-39

# 40-49

# 50-59

# 60+

# 3. Years practicing as a nurse

# < 5 years

# 5- 10 years

# 11-15 years

# 16- 20 years

# >20 years

# 4. Years working as a nurse in PICU

# < 5 years

# 5- 10 years

# 11-15 years

# 16- 20 years

# >20 years

# 5. Number of family meetings you have attended where there was discussion of limiting/withdrawing therapy or DNR

# <5

# 5-10

# 10-15

# >15

# Page 2: Intensivists role

1. *Threshold for approaching a family:* **Strongly Agree Neutral Disagree Strongly**

## Agree Disagree

1. Each of the PICU Intensivists has the same threshold for approaching a

family to suggest a limiting/withdrawing life support or DNR order. **    **

1. This threshold is too high with some Intensivists.

(i.e. the discussion occurs too late) **    **

1. This threshold is too low with some Intensivists.

(i.e. the discussion occurs too early) **    **

1. *Family contribution to decision:*
2. Each intensivist allows the same amount of family contribution to the

decision regarding limiting/withdrawing life support or DNR orders.  **    **

1. Too much family influence is allowed with some Intensivists. **    **
2. Too little family influence is allowed with some Intensivists. **    **
3. *Unilateral decision:*

a. A PICU intensivist has withdrawn/limited therapy without having a

discussion with the family. **    **

1. This occurs often. **    **

c. Each intensivist has the same threshold for withdrawing/limiting

therapy without having a discussion with the family. **    **

1. *Survival vs Quality of Life:*

a. Each intensivist has the same threshold of the patient’s chance for survival

when making a decision to limit/withdraw therapy **   **  ****

b. Each intensivist has the same threshold of the patient’s projected

quality of life when making a decision to limit/withdraw therapy. **    **

c. The threshold to suggest to limit/withdraw therapy based on chance <**1% 5% 10% 15% ≥20%**

of survival differs between intensivists by: **    **

d. The threshold to suggest to limit/withdraw therapy based on projected **<1% 5% 10% 15% ≥20%**

quality of life differs between intensivists by: **    **

### Page 3: The Nurse’s role

1. *Your own child:*
2. Assume your child was in the PICU and the intensivist on service

approached you to recommend a limiting/withdrawing life support

or DNR order. You would have equal confidence accepting this

recommendation from each intensivist. **    **

1. You would have confidence in the intensivist’s opinion to

limit/withdraw life support only in certain situations.  **    **

Please explain:
