# Supplementary material for: Variability in the pediatric intensivists' threshold for withdrawal/limitation of life support as perceived by bedside nurses: a multicenter survey study
Source: Ann Intensive Care. 2011 Aug 8;1:31. doi: 10.1186/2110-5820-1-31 (PMC3224498; doi:10.1186/2110-5820-1-31)
Supplement: Additional file 3 — Representative written comments to the instruction "please explain" after questions about confidence in intensivist decisions for the nurse's hypothetical own child. Table [file 2110-5820-1-31-S3.DOC]

**Variability in the pediatric intensivists’ threshold for withdrawal/limitation of life support as perceived by bedside nurses: a multicenter survey study**

**Journal Name:** Annals of Intensive Care

**Authors:** Colleen S Gresiuk RN, BScN^1^; and Ari R Joffe MD, FRCPC^1,2^

**Affiliations:** ^1^University of Alberta, ^1^Stollery Children’s Hospital, and ^2^The John Dossetor Health Ethics Center; Edmonton, Alberta, Canada.

**Corresponding author:** Ari R Joffe MD; Email: [ari.joffe@albertahealthservices.ca](mailto:ari.joffe@albertahealthservices.ca)

**Additional File 3**: Representative written comments to the instruction “please explain” after questions about confidence in intensivist decisions for the nurse’s hypothetical own child.

| **Category of Comment** | **Representative examples** |
| --- | --- |
| **Confidence Level in the intensivists’ recommendations.** | |
| **Total confidence in the intensivists’ recommendations** | I would be confident that the information given to me was accurate and thoroughly reviewed before they would recommend withdrawing life support. |
|  | I have never felt that any intensivist has limited the care offered to a patient prematurely, nor do I feel that certain intensivists hold less reliability in their recommendations. |
|  | I fully trust all of the intensivists’ decisions with respect to DNR. It is a difficult decision and I trust them to make the right call. |
| **Confidence in the intensivists’ recommendations only in certain situations** | Cultural background and beliefs of intensivists play important role in this decision, in addition to his medical skills. I might not have the same point of view as the doctor, therefore I would do a lot of research and take different opinions and consult different doctors before taking or thinking of such a decision no matter whom the intensivist is. |
|  | If it was my child, I would like to review with them the evidence i.e. EEG, apnea tests, neuro exam and speak with a neurologist as well (second opinion). |
|  | If my child was in the ICU I would wait to get a variety of opinions on prognosis to help inform my decision making and I would take into account the variation I see between the style of practice of each MD. |
|  | Depends upon what evidence the intensivist is basing his/her opinion on, years of experience, and his/her definition of “quality of life”. |
|  | At times I may not agree with intensivists perceptions of quality of life. |
|  | It seems that the idea of “quality of life” is not only fluid between practitioners, but between situations with the same intensivist. |
|  | It would depend on my values and beliefs versus the intensivist i.e. quality of life from my point of view versus his/hers. |
| **Lack of confidence in the intensivists’ recommendations** | My child was in the ICU and an intensivist did approach me re withdrawing care, and I refused withdrawal. My daughter got well and went home 3 weeks later. |
|  | Depends on which intensivist and why we are withdrawing care. |
|  | Depending upon which intensivist is on service I would feel more comfortable in the intensivist’s opinion because some intensivists don’t approach these situations well/frequently and based on working with the docs, there are certain ones who I am more comfortable with. |
|  | I’ve seen a very varied approach to end of life/withdrawing of support between physicians. I see intensivists not getting along a lot. |
|  | I have seen where one doc has given a grim picture and then the next one on has painted it in rosier light, so you are not sure which one to believe. |
|  | Depends on who the intensivist is, who I trust. |
|  | I have differing levels of confidence in the staff intensivist’s clinical judgment in the PICU. It would depend on who the recommendation was coming from. |
| **General comments on how the decisions should be made** | |
| **Need for multidisciplinary involvement in making W/L decisions** | I would also want other specialty areas input as well, i.e. for a head injury have neuro involved. |
|  | All disciplines involved in the care of the child have met and all are in agreement before this suggestion is presented to the patient’s family. |
|  | This decision is not made solely by one person- it is often discussed between many medical persons. |
|  | I think that each intensivist has a certain expertise in their field, so I would expect them to consult the “experts” before approaching me with a DNR to ensure no stone was left unturned. |
| **The timing of the recommendation is delayed too long** | The possibility of intensivists waiting too long to make important treatment decisions is more common. |
|  | Because we wait too long to have these talks with family then when the decision is made to withdraw care it is too late. |
|  | I often find we prolong patient’s lives when death/withdrawal is inevitable. |
|  | Some seem to avoid these conversations at all costs even in blatantly futile cases. |
| **Other comments** | I strongly believe a parent should never have to make this decision to stop treatment or to “pull” life support, ever, therefore the need for an ethics committee is so important. |
|  | It would take me a long time to get there (to agree). |
|  | It is all in the approach taken by the intensivist, i.e. some are better at talking with families than others. |

DNR: do not resuscitate; EEG: electroencephalogram; MD: medical doctor; PICU: pediatric intensive care unit.
